# Very low birth weight infant outcomes in a resource-limited setting: a five-year follow-up study

**DOI:** 10.3389/fped.2025.1581033

**Published:** 2025-05-01

**Authors:** Windhi Kresnawati, Peter John Pandie, Rinawati Rohsiswatmo

**Affiliations:** ^1^ Department of Child Health, Gatot Soebroto Military Hospital, Jakarta, Indonesia; ^2^ Department of Child Health, Biak General Hospital, Papua, Indonesia; ^3^ Department of Child Health, Cipto Mangunkusumo Hospital, Jakarta, Indonesia

**Keywords:** preterm neonates, survival rate, low birth weight, developmental impairment, growth

## Abstract

**Background:**

Preterm birth and very low birth weight (VLBW) remain major contributors to neonatal morbidity and mortality, particularly in low-income settings such as Indonesia, where healthcare resources are limited. In response, the Ministry of Health introduced mentoring programs in 2014, followed by intensive neonatal training initiatives in 2015. These interventions substantially improved survival rates for LBW infants however, they face significant growth and developmental challenges. This study aims to investigate the growth and development of VLBW infants in remote area at five years of age.

**Method:**

Data were collected retrospectively between September 2021 and May 2022 from children born between 2016 and 2017 with VLBW (<1,500 grams) at Biak Regional Hospital, Indonesia. Growth parameters, including stunting and wasting and developmental outcomes such as cerebral palsy, blindness, and developmental impairment or delays were assessed after 5 years of age for follow up assessment.

**Results:**

Among 78 identified infants with VLBW, 54 infants (69.2%) with a gestational age of <34 weeks were discharged alive between 2016 and 2017. Their gestational ages ranged from 27 to 33 weeks, with birth weights between 625 and 1,400 grams. Overall, 12 infants died before reaching one year of age while five died after one year. The 1-year survival rate was 77.8%, while 5-year survival rate was 68.5%. The incidences of stunting, wasting, cerebral palsy, and blindness were 32.1%, 46.4%, 21.4%, and 10.7%, respectively.

**Conclusion:**

The high prevalence of growth and developmental impairments highlights the need for sustained multidisciplinary efforts to improve long-term outcomes for VLBW infants. In resource-limited settings, the focus should extend beyond survival to ensure optimal growth and development of the children.

## Introduction

Preterm birth remains a global health concern, affecting approximately 9.9% of live births worldwide annually (1). It is defined as birth of a baby before 37 weeks of gestation and is further sub-categorized as: extremely preterm (<28 weeks), very preterm (28–<32 weeks), and moderate to late preterm (32–37 weeks) (2). According to the World Health Organization (WHO), an estimated 13.4 million infants were born preterm in 2020 (3). In Indonesia, preterm birth accounted for 29.5% of all births in 2018, ranking the country 5th globally for the highest number of preterm births (4). Approximately 900,000 deaths occurred among children under five years old in 2019 due to inadequate access to cost-effective, essential care such as thermal regulation, breastfeeding support, and basic management of infections and respiratory distress (3, 5). In low-and middle-income countries, suboptimal use of medical technology has contributed to a growing burden of disabilities among preterm survivors (6).

Preterm birth is strongly associated with low birth weight (LBW) (<2,500 g), a major contributor to neonatal mortality and morbidity (7). LBW results from intrauterine growth restriction, prematurity, or both and is linked to higher risks of fetal and neonatal mortality, poor growth, cognitive delays, and non-communicable diseases later in life (8). In Indonesia, LBW and/or prematurity accounted for 28.3% of neonatal deaths, followed by asphyxia at 26.3% (9, 10). However, advances in perinatal care have improved survival rates and addressed postnatal growth challenges and developmental delays, particularly for very LBW (VLBW) infants (<1,500 g) (11). In 2022, WHO released updated guidelines on preterm infant care, emphasizing evidence-based interventions such as immediate kangaroo mother care, early breastfeeding initiation, continuous positive airway pressure (CPAP), and the administration of caffeine for respiratory support to reduce mortality in preterm and low-birth-weight infants (12).

Biak, a small island in Papua, Indonesia, represents a resource-limited setting where preterm infant outcomes remain challenging (13). Papua and West Papua have one of the highest neonatal mortality rates in Indonesia (27–35 per 1,000 live births) (14). The Perinatology Unit of Biak Regional Hospital was established in 2011 as an independent facility separate from the pediatric care unit. However, the neonatal mortality rate remains high (35–37 per 1,000 live births) due to limited medical resources and workforce shortages. To address these challenges, the Indonesian Ministry of Health introduced a mentoring program in 2014, followed by comprehensive neonatal resuscitation and infection control training in 2015 (13). These initiatives resulted in a significant improvement in survival rates for VLBW infants, increasing from 0% to 64% (13). Despite these advancements, uncertainties persist regarding the long-term outcomes of preterm infants discharged from Biak Regional Hospital (15). The hospital faces persistent limitations, including the absence of ophthalmologists and ENT specialists, inadequate respiratory support equipment, suboptimal parenteral nutrition management, and insufficient monitoring of blood gas levels and oxygen saturation, thereby, elevating the risk of growth impairments and developmental delays. Additionally, socioeconomic factors such as poverty, low parental education, limited healthcare accessibility, and geographical barriers further contribute to post-discharge morbidity and mortality among preterm infants (16).

Based on this, this study aims to evaluate the long-term survival, growth and developmental outcomes of VLBW infants over a period of five years in a resource-limited setting.

## Materials and methods

### Study population

This observational study included all the VLBW infants (<1,500 grams) born before 34 complete gestational weeks between 2016 and 2017 who were admitted to the Perinatology Unit of Biak Regional Hospital, Papua. These infants were monitored and followed up till the age of 5 years between September 2021 and May 2022. Gestational age was determined using the mother's last menstrual period or first-trimester ultrasound examination. Ethical approval was obtained from the Institutional ethics committee at the Biak Regional Hospital, Papua, Indonesia. Informed consent was obtained from all parents or guardians prior to participation in the study.

### Study objectives

The primary objective of the study was to determine one-year and five-year survival rate of VLBW infants in resource- limited setting. Additionally, the study assessed the proportion of growth and developmental disorders at five years of age and examined how neonatal care quality (oxygen administration) affected developmental outcomes later in life. The study also identified factors associated with survival and developmental impairment, including gestational age, birth weight, gender, residence location, and caregiving environment. Lastly, the study compared survivors and non-survivors based on demographic and clinical characteristics to understand potential risk factors influencing survival.

### Data collection

This study employed a retrospective and cross-sectional data collection approach. Neonatal and demographic data were collected from hospital medical records, including birth weight, gender, gestational age, oxygen exposure (FiO2 levels and duration), length of hospital stay, and discharge status. All the neonates were then traced through their provided addresses on the medical records, and verified using local village head officer data. The patients were followed up after one year to assess survival rates and again at five years of age for developmental and growth assessments. At 5 years, follow up assessments were conducted either via video call or through direct examination by trained medical professionals. Two nurses and one general practitioner were trained to conduct anthropometric examination and Denver developmental screening test (DDST), under the supervision of a pediatrician. During video call assessments, caregivers were guided by these trained professionals to assist in evaluating motor function and visual impairments. Caregivers were instructed on these simple tests to assess vision or perform basic motor tasks.

### Growth assessment

Anthropometric measurements followed the WHO growth chart, which involves assessing key physical parameters such as weight, height, and head circumference. These measurements were plotted on standardized WHO growth charts to evaluate nutritional status and growth patterns relative to a healthy reference population (17, 18). Growth and nutritional status were categorized using standard WHO definitions: stunting was classified as a length-for-age Z-score (LAZ) <−2 SD and wasting as a weight-for-length Z-score <−2 SD (19, 20). The age of children at the time of assessment was five years.

### Developmental assessment

DDST is a standardized tool designed to screen for potential developmental delays in infants and preschool-aged children. It assesses four key functional domains: gross motor skills, language development, fine motor-adaptive skills, and personal-social interactions (21, 22). As per the manual, the standard outcomes of DDST were interpreted as “normal” or “suspect”. If the result was categorized as “suspect,” the child was referred to a pediatrician for further evaluation and diagnosis, such as cerebral palsy (CP), blindness, or other developmental conditions. In case of inconclusive results, the test was repeated. Suspicion of developmental delay was defined as failing in two or more items in a single domain or one item in two or more domains.

Further, cerebral palsy is defined as the presence of motor impairments or neurological deficits. The presence or absence of CP was clinically assessed in infants and children without categorizing the severity. Blindness is defined as the inability to see with one eye, assessed by covering one of the eyes. No formal ophthalmologic examinations were performed but data on blindness were collected through self-reports or caregiver reported observations.

### Factors influencing developmental outcomes

Patients included in the study were categorized based on their residential location relative to the hospital catchment area and those who were cared for by their biological parents or extended families to assess their relationship with survival rates. The hospital catchment area was defined as the Biak Kota District. Patients living far from the hospital were considered residing outside the hospital catchment area. The impact of residence location on the survival rates of the children was assessed. Further, retrospective data were obtained from medical records to evaluate oxygen exposure. Oxygen administration was classified as high when the FiO_2_ exceeded 40%, and as prolonged if it lasted more than 96 hours. The association between oxygen exposure and developmental impairment was assessed.

### Statistical analysis

Statistical analyses were conducted using SPSS software version 20.0 for Windows (SPSS Inc., Chicago, IL, USA). Categorical variables including the incidence of cerebral palsy, stunting, and wasting, blindness were presented in numbers and percentages. We primarily checked the normality of the data using Kolmogorov–Smirnov test. Based on normality, the categorical data was analyzed using chi-square test. Continuous variables were assessed using Student's *t*-test. For correlation analysis, the Pearson correlation coefficient (r) test was used to assess the association between birth weight and developmental quotient. A *p*-value of <0.05 was considered statistically significant.

## Results

### Survival outcomes

**Figure 1:**
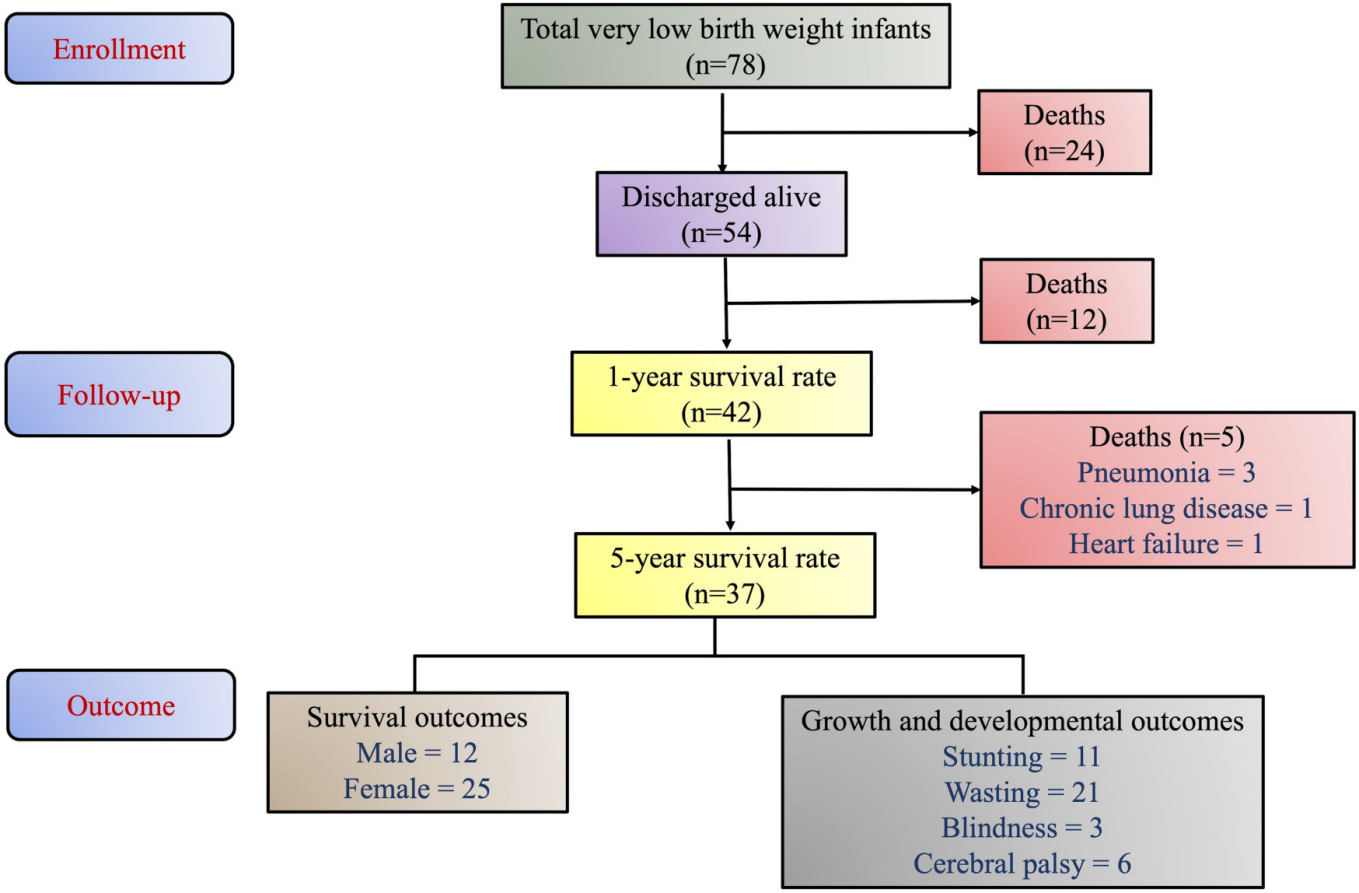
Patient flowchart.

A total of 78 neonates with a VLBW below 1,500 grams were born at Biak Regional Hospital between 2016 and 2017. Of these, 54 (69.2%) were discharged alive with the male-to-female ratio of 24:30. Birth weights ranged from a minimum of 625 grams (26 weeks gestational age) to a maximum of 1,480 grams (33 weeks gestational age). A five-year follow-up data between 2021 and 2022 revealed that 37 out of the 54 discharged infants (68.5%) survived while the one-year survival rate was 77.8% (42/54). Overall, 12 deaths occurred within the first year and five deaths were recorded after one year. The cause of death of five children who passed away after one year was pneumonia (3 patients), chronic lung disease (1 patient), and heart failure (1 patient). The patient flowchart is presented in Figure 1.

Further, the survival influencing factors were assessed by comparing survivors and non-survivors in terms of gender, average body weight, and average gestational age. We observed a statistically significant difference in average gestational age and male-to-female ratio (*p* < 0.05). However, no significant difference was found in average body weight of survivors and non-survivors (*p* = 0.06). Further, we assessed the impact of residence location on survival rates. The survival rates of children living within the hospital catchment area (Biak Kota District) were significantly higher than those residing in remote areas (*n* = 37 vs. 17, *χ*^2^ = 4.96, *p* = 0.02). A detailed comparative data of survivors and non-survivors is given in Table 1.

**Table 1:** Comparative data of survivors and non-survivors.

Parameters [*n* (%) mean ± SD]	Survivors (*n* = 37)	Non-survivors (*n* = 17)	*χ* ^2^	*p*-value
Gender			6.86	**0** **.** **009**
Male	12 (32.4)	12 (70.6)		
Female	25 (67.6)	5 (29.4)		
Mode of delivery			0.01	0.98
Vaginal delivery	26 (70.3)	12 (70.6)		
C-section	11 (29.7)	5 (29.4)		
Average body weight, g	1,255.4 ± 181.8	1,174.1 ± 191.2	–	0.13
Average gestational age, weeks	30.9 ± 2.0	29.5 ± 2.1	–	**0**.**02**
Length of hospital stay, days	20.0 ± 14.6	19.9 ± 20.1	–	0.97
Hospital catchment area:			4.96	**0**.**03**
Yes	25 (67.6)	6 (35.3)		
No	12 (32.4)	11 (64.7)		

*n*, number of patients; %, percentage; SD, standard deviation; g, grams. Statistical analysis by *t*-test and chi-square test.

Bold values are statistically significant (*p* < 0.05).

### Growth and developmental outcomes

Among the 37 surviving children, the prevalence of growth and developmental impairments was assessed. Stunting was observed in 29.7% of cases (*n* = 11), while wasting was seen in 56.7% (*n* = 21). Additionally, blindness and cerebral palsy were present in 8% (*n* = 3) and 16% (*n* = 6) of cases, respectively.

### Factors influencing developmental outcomes

Further, children with and without developmental impairment were compared in terms of gestational age, birth weight, and oxygen exposure. A significant association was found between prolonged, high-concentration oxygen exposure and developmental impairment (*p* = 0.02) however, no difference was observed for gestational age in children with and without developmental impairment (*p* = 0.07), However, a significant increase in birth weight was observed in infants without developmental impairment (1,292.8 ± 153.5) as compared to affected infants (1,154.5 ± 220.7; *p* = 0.04) (Table 2). Moreover, when correlation analysis was conducted between birth weight and developmental impairment, a significant and positive correlation was observed (R = 0.34, *p* = 0.04), suggesting birth weight affects the occurrence of developmental disorders.

**Table 2:** Relationship between developmental impairment and gestational age, birth weight and oxygen delivery.

Parameters [*n* (%) mean ± SD]	Developmental impairment (*n* = 10)	No developmental impairment (*n* = 27)	χ^2^	*p*-value
Average gestational age, weeks	30.1 ± 2.1	31.2 ± 1.9	–	0.14
Average birth weight, g	1,154.5 ± 220.7	1,292.8 ± 153.5	–	**0** **.** **04**
Oxygen delivery			5.39	**0**.**02**
High	8 (80.0)	10 (37.0)		
No	2 (20.0)	17 (63.0)		

*n*, number of patients; %, percentage; SD, standard deviation; g, grams. Statistical analysis by *t*-test and chi-square test.

Bold values are statistically significant (*p* < 0.05).

In this study, 25.9% of children were not raised by their biological parents but by extended family members such as grandparents or aunts. When the survival rate, nutritional status, or developmental outcomes were compared between children raised by biological parents and those cared for by extended family members, no significant differences were observed (*p* < 0.05). The relationship between care taker and survival rate, malnutrition and developmental problem is shown in Table 3.

**Table 3:** Relationship between care taker and survival rate, malnutrition and developmental problem.

Parameters (infant raised by) [*n* (%)]	Biological parents	Extended family	χ^2^	*p*-value
Survival	(*n* = 40)	(*n* = 14)	1.13	0.29
Survivors	29 (72.5)	8 (57.1)		
Non-survivors	11 (27.5)	6 (42.9)		
Nutrition	(*n* = 29)	(*n* = 8)	1.38	0.24
Malnutrition	15 (51.7)	6 (75.0)		
Good nutrition	14 (48.3)	2 (25.0)		
Developmental impairment	(*n* = 29)	(*n* = 8)	2.73	0.10
Yes	6 (20.7)	4 (50.0)		
No	23 (79.3)	4 (50.0)		

*n*, number of patients; %, percentage. Statistical analysis by chi-square test.

## Discussion

In Biak, the rate of premature births has been increasing annually, driven by risk factors such as teenage mothers and the presence of maternal diseases like anemia and malaria (23). For instance, the Biak Regional Hospital reported 108 premature infants in 2011, which increased to 271 (22% of total live births) by 2014. Premature infants exhibited a range of health challenges that reflect the quality of neonatal care, especially in areas with limited resources (24). In Indonesia, there is a strong emphasis on expanding hospital services, adding specialist doctors, and enhancing medical infrastructure (25). However, community-based health promotion play a significant role in reducing child morbidity and mortality. This study is a continuation of a previously published quality improvement program in the NICU of Biak Hospital, which significantly reduced newborn mortality who previously had limited chances of survival (13). As survival improved, the need to address long-term outcomes such as growth and developmental impairments became increasingly apparent. The present study provides essential insights into outcomes for premature infants in a remote setting, specifically focusing on the survival rates, disabilities, and health challenges faced by VLBW infants in Biak, Papua.

In our study, 1-year and 5-year survival rates of VLBW infants were 77.8% and 68.5%. In a study, Tamene et al. (2020) assessed the survival rate of preterm neonates admitted to Felege Hiwot Specialized Hospital, Ethiopia and observed survival rate of 75% (26). Another study by Mocking et al. (2023), the authors determined the survival rates and outcomes of preterm births up to six weeks of corrected age in Ghana and found 81.5% of survival rate after 6 weeks (27). These studies are in concordance to our findings of 1-year survival rates. Compared to the high income countries, where extremely premature infants (gestational age of 27 weeks) had a survival rate ranging from 75% to 98% (28). This gap highlights the ongoing challenges of providing optimal care for preterm infants particularly in low-resource settings where medical interventions are limited. Furthermore, the survival rates were significantly higher for the patient residing in hospital catchment area as compared to residing outside. This may be attributed to the increased number of primary health centers (puskesmas) in Papua by more than 50% over a decade (2007–2017) (29). However, the doctor-to-health center ratio in Papua is 0.9, significantly lower compared to major cities such as Bali (3.8) and Jakarta (4.9) (29), contributing to high mortality rate in children under age of five.

Table 1 demonstrated key characteristic differences between survivors and non-survivors at baseline. Gender was significantly associated with survival (*p* = 0.009), with a higher proportion of non-survivors being male (70.6%) compared to survivors (32.4%). In a study, Qurashi et al. (2025) examined the impact of gender on survival trends of extremely LBW infants and found that female infants have a higher survival rate than males (30). This is in concordance with our findings. Further, the risk of neonatal mortality increases with lower gestational age and decreased birth weight (31). We also observed that gestational age was significantly lower in non-survivors (29.5 ± 2.1 weeks) compared to survivors (30.9 ± 2.0 weeks, *p* = 0.02), indicating that earlier gestational age increases mortality risk. Another notable finding was the impact of hospital catchment area (*p* = 0.03); infants residing within the hospital catchment area had a higher survival rate (67.6%) compared to those from outside the area (35.3%). This suggests that proximity to healthcare facilities improves neonatal outcomes.

Next, high oxygen levels have been associated with neurodevelopmental impairments in preterm infants during early care (32). We also observed that high-concentration oxygen exposure was significantly associated with developmental impairment (*p* = 0.02), with 80% of children with impairments having received high oxygen concentrations compared to 37% of those without impairments (Table 2). This finding highlights the risks of prolonged oxygen therapy in preterm infants, particularly in settings lacking adequate respiratory support technologies.

Beyond survival, the caregiving environment plays a crucial role in long-term outcomes for premature infants (33). A significant proportion (25.9%) were raised by extended family members, such as grandparents or aunts. Several factors contributed to this arrangement, including maternal death during childbirth or from other illnesses, the mother being of school age, employment-related separation, or the biological parents’ inability to provide care due to economic constraints or lack of family readiness. Notably, this did not impact the child survival rate, nutritional status, or developmental disorders, reflecting the parents’ careful decision-making in selecting suitable caregivers. This caregiving model contrasts with urban settings where children are predominantly cared for by biological parents or hired caregivers (34). According to 2018 data from the Indonesian Child Protection Commission (KPAI), 75% of children in Jakarta are cared for by babysitters or daycare services, while 14% are under the care of grandparents (35).

As observed in the study, the rate of cerebral palsy among VLBW infants was notably high, at 21.4%, which is in line with previous research highlighting the increased risk of cerebral palsy in preterm populations (36). This is due to several factors, including the high incidence of intracranial bleeding, periventricular leukomalacia, and non-optimal brain development due to premature birth (37). Moreover, the impact of malnutrition on cognitive and developmental outcomes is an area of concern, as the study highlighted that 46.4% of patients were malnourished, with a significant proportion of them experiencing stunting.

The rate of blindness (10.7%) is considerably lower than those seen in other studies, such as Egypt and other developed countries (38). This lower blindness rate could be attributed to limited access to comprehensive eye exams, which may have resulted in only severe cases of visual impairment being identified. This is an important observation, as it suggests that while neonatal care programs in Biak may be successful in preventing more severe cases of retinopathy of prematurity (ROP), there may be a need for better screening and diagnosis to capture all instances of visual impairment.

Further, a significant association was found between high-concentration and prolonged oxygen exposure and developmental disorders later in life. High-concentration oxygen administration was caused by several factors: lack of CPAP and oxygen blenders, which led to the use of nasal cannula oxygen delivery in patient overload conditions, unavailability of adequate invasive respiratory support equipment, such as mechanical ventilators or high-frequency oscillatory ventilators (HFO), resulting in increased oxygen fraction as the primary intervention for ventilation and oxygenation, and absence of surfactant therapy for preterm infants, leading to prolonged use of respiratory support in cases of hyaline membrane disease (39).

*Limitations and Future directions:* This study has several limitations. First, it was conducted in a single remote region (Biak, Papua), which may limit the generalizability of the findings to other settings with different healthcare infrastructures and population demographics. Secondly, the survival outcomes and long-term complications were assessed retrospectively, relying on available medical records and self-reported follow-up data. Thirdly, the potential confounding factors such as variations in maternal health, socioeconomic status, and healthcare access, were not assessed which may influence preterm survival and long-term outcomes.

Based on the findings and limitations of the study, future research should focus on larger and multicentric studies to understand variations in neonatal care and outcomes across different healthcare settings. The significant association between high-concentration oxygen exposure and developmental impairment highlights the need for improved respiratory support strategies, such as better access to CPAP and oxygen blenders. Moreover, the observed disparities in survival rates based on proximity to healthcare facilities emphasize the importance of strengthening neonatal care networks and improving referral systems for high-risk infants. Prospective studies incorporating standardized follow-up protocols would also improve the accuracy of long-term developmental assessments. Additionally, investigating the impact of specific neonatal interventions, such as oxygen therapy protocols and nutritional support programs, could help refine best practices for managing preterm infants in resource-limited settings. Recognizing the high prevalence of cerebral palsy and malnutrition among VLBW infants underscores the necessity of early intervention programs, including physical therapy and nutritional support, to improve long-term outcomes. Strengthening neonatal follow-up services and incorporating developmental screenings into routine pediatric care could aid in the early identification and management of neurodevelopmental impairments, ultimately improving the quality of life for preterm infants.

## Conclusion

This study examined the survival rates, growth outcomes, and long-term complications of VLBW preterm infants in a remote setting. The one-year survival rate was 77.8%, while the five-year survival rate was 68.5%, highlighting the ongoing risk of mortality beyond the neonatal period. Accessibility to healthcare facilities was a significant factor influencing survival, with infants residing within the hospital's catchment area experiencing better outcomes than those in remote locations. Despite improved survival, a considerable proportion of surviving children experienced growth impairments, with stunting observed in 29.7% and wasting in 56.7%. Additionally, developmental disorders such as cerebral palsy (16%) and blindness (8%) were prevalent, reinforcing the long-term health challenges faced by preterm infants. High-concentration oxygen exposure was significantly associated with developmental impairments, emphasizing the need for optimized respiratory support strategies. Moreover, birth weight was positively correlated with better neurodevelopmental outcomes. Overall, this study highlights the critical need for improved neonatal care and nutritional interventions for VLBW infants after discharge in remote areas such as Biak. The findings suggest that providing early nutritional support, as well as addressing the limitations in medical interventions, may significantly improve the health outcomes for preterm infants in such settings.

## Data Availability

The raw data supporting the conclusions of this article will be made available by the authors, without undue reservation.
